# High Efficacy and Drug Synergy of HDAC6-Selective Inhibitor NN-429 in Natural Killer (NK)/T-Cell Lymphoma

**DOI:** 10.3390/ph15111321

**Published:** 2022-10-26

**Authors:** Harsimran Kaur Garcha, Nabanita Nawar, Helena Sorger, Fettah Erdogan, Myint Myat Khine Aung, Abootaleb Sedighi, Pimyupa Manaswiyoungkul, Hyuk-Soo Seo, Susann Schönefeldt, Daniel Pölöske, Sirano Dhe-Paganon, Heidi A. Neubauer, Satu M. Mustjoki, Marco Herling, Elvin D. de Araujo, Richard Moriggl, Patrick T. Gunning

**Affiliations:** 1 Department of Chemical and Physical Sciences, University of Toronto Mississauga, 3359 Mississauga Road, Mississauga, ON L5L 1C6, Canada; 2 Department of Chemistry, University of Toronto, 80 St. George Street, Toronto, ON M5S 3H6, Canada; 3 Institute of Animal Breeding and Genetics, University of Veterinary Medicine Vienna, 1210 Vienna, Austria; 4 Department of Cancer Biology, Dana-Farber Cancer Institute, Boston, MA 02215, USA; 5 Department of Biological Chemistry and Molecular Pharmacology, Harvard Medical School, Boston, MA 02215, USA; 6 Translational Immunology Research Program and Department of Clinical Chemistry and Hematology, University of Helsinki, 00014 Helsinki, Finland; 7 Hematology Research Unit, Helsinki University Hospital Comprehensive Cancer Center, 00290 Helsinki, Finland; 8 iCAN Digital Precision Cancer Medicine Flagship, 00014 Helsinki, Finland; 9 Department of Hematology, Cellular Therapy, and Hemostaseology, University of Leipzig, 04109 Leipzig, Germany

**Keywords:** NKTCL, HDAC6, synergy, combination treatment, small molecule inhibitor

## Abstract

NK/T-cell lymphoma (NKTCL) and γδ T-cell non-Hodgkin lymphomas (γδ T-NHL) are highly aggressive lymphomas that lack rationally designed therapies and rely on repurposed chemotherapeutics from other hematological cancers. Histone deacetylases (HDACs) have been targeted in a range of malignancies, including T-cell lymphomas. This study represents exploratory findings of HDAC6 inhibition in NKTCL and γδ T-NHL through a second-generation inhibitor NN-429. With nanomolar in vitro HDAC6 potency and high in vitro and in cellulo selectivity for HDAC6, NN-429 also exhibited long residence time and improved pharmacokinetic properties in contrast to older generation inhibitors. Following unique selective cytotoxicity towards γδ T-NHL and NKTCL, NN-429 demonstrated a synergistic relationship with the clinical agent etoposide and potential synergies with doxorubicin, cytarabine, and SNS-032 in these disease models, opening an avenue for combination treatment strategies.

## 1. Introduction

Peripheral T-cell lymphomas (PTCLs) represent a heterogeneous group of rare diseases, of which many are associated with diagnostic complexities and poor patient survival, coupled to a lack of targeted therapies [1,2]. Some of the most aggressive entities with the poorest clinical outcomes encompass the γδ T-cell non-Hodgkin lymphomas (γδ T-NHL), including hepatosplenic T-cell lymphoma (HSTL) and monomorphic epitheliotropic intestinal T-cell lymphoma (MEITL) as well as NK/T-cell lymphomas (NKTCLs) [3]. With widely varying clinical presentations, there are a lack of efficient therapies that induce long-lasting profound remission in γδ T-NHL and NKTCL [4] The current strategies in both disease groups are predominantly non-targeted conventional or high-dose chemotherapy approaches, often repurposed from B-cell lymphoma protocols. However, they often fail in long-term tumor control and are associated with considerable toxicities. Current commonly used chemotherapeutic protocols for γδ T-NHL and NKTCL include anthracycline-containing regimens such as CHOP (cyclophosphamide, doxorubicin, vincristine, and prednisone), or combinations of agents such as etoposide, gemcitabine, and platin-derivatives. A persisting problem in many PTCL is primary or early acquired [1,5,6] chemotherapy resistance [6]. The most beneficial outcome of a chemotherapy-based first-line treatment of a PTCL is to achieve a complete remission (CR), which is then followed by a consolidating high-dose chemotherapy with autologous or allogeneic hematopoietic stem cell transplantation in a curative attempt [1,2,6] However, only about two-thirds of chemo-induced patients reach such a first CR and only 40–50% of PTCL patients are transplant-eligible in general [4,6,7,8,9].

The lack of targeted therapeutics for PTCLs is not only fueled by disease heterogeneity and molecular complexities, but also by the challenges of patient recruitment and absence of robust, translatable pre-clinical disease models. The rarity of γδ T-NHL and NKTCLs makes performing a clinical trial so arduous that trials are infrequently dedicated solely to these disease entities. A more common phenomenon is the integration of multiple heterogeneous PTCL patient populations into a single trial [6].

Current attractive investigational agents for subsets of PTCL include JAK inhibitors, DNA-demethylating agents, and particularly HDAC inhibitors (romidepsin, chidamide, belinostat) [6,10]. The pan-HDAC inhibitor chidamide combined with the clinical agents etoposide, carboplatin, and ifosfamide, has shown improvements for γδ T-cell lymphoma patients with CRs lasting up to 9 months, while standard chemotherapy failed to disrupt disease progression [9]. HDAC inhibition has shown more effectiveness in T-cell malignancies over B-cell malignancies, with the ability to overcome chemo-resistance [11,12]. To date four HDAC inhibitors (SAHA, belinostat, romidepsin, and panobinostat) have been approved by the FDA for the treatment of hematological malignancies. All result in adverse side effects in the clinic due to non-selective inhibition of multiple HDAC isoforms, warranting the development of isozyme-selective inhibitors [13].

Histone deacetylase (HDAC) enzymes catalyze the deacetylation of histone and non-histone proteins. The HDAC family consists of 18 human proteins, 11 of which are Zn^2+^-dependent metalloenzymes and 7 are NAD^+^-dependent (known as sirtuins). HDACs are categorized into 4 groups based on homology to their yeast analogs: class I (HDAC1, HDAC2, HDAC3, HDAC8), class II (class IIa: HDAC4, HDAC5, HDAC7, HDAC9; class Iib: HDAC6, HDAC10), class III (sirtuins) and class IV (HDAC11) [14]. HDAC6 is the largest protein of the HDAC family with 1215 amino acids, two catalytic domains (CD1 and CD2) and a unique C-terminal zinc-finger ubiquitin-binding domain (ZnF-UBP) [13].

Out of the family of 11 HDAC isozymes, HDAC6 has emerged as a highly attractive and safe target in drug discovery, because of its imperative roles in cellular function and survival, and well-tolerated loss of function when inhibited. The cytoplasmic clients of HDAC6 include an extensive substrate repertoire such as α-tubulin, cortactin, HSP90, tau and peroxiredoxins. As such, HDAC6 is involved in cellular processes of cell migration, cell mobility, stress response, autophagy protein degradation, and intracellular trafficking [15,16,17,18,19]. Aberrant HDAC6 activity has been associated with cancer progression, neurodegenerative diseases, and inflammatory disorders [20,21]. HDAC6 is overexpressed in multiple cancers cell lines such as ovarian cancer cells, primary oral squamous cells, primary acute myeloid leukemia blasts and myeloblastic cell lines [22]. In MCF-7 cells, HDAC6 expression may be upregulated by estrogen and that could influence the metastasis of breast cancer [23]. The upregulation of HDAC6 in a range of tumors suggests a pleiotropic role in cancer progression.

For the last decade, efforts have been made to target the HDAC family of enzymes for therapeutic intervention. HDAC inhibitors typically consist of a cap group which interacts with the surface of the enzyme, a zinc binding group (ZBG) that interacts with the zinc ion in the catalytic pocket, and a linker between these two moieties [24]. The most common ZBG used is the hydroxamic acid, which is capable of chelating metal ions such as Zn(II) and Fe(III). This chelating ability of hydroxamic acids has been exploited to develop inhibitors for several of the metal-bearing HDAC enzymes [24,25,26]. Currently, four pan-HDAC inhibitors (SAHA, romidepsin, belinostat, and panobinostat) have been approved by the FDA for the treatment of T-cell lymphoma or multiple myeloma, out of which 3 of them include hydroxamic acids. However, all four drugs present broad spectrum HDAC activity, resulting in various adverse effects that limit their application to relapsed cancer patients. Thus, there is escalated interest in the development of isozyme selective HDAC inhibitors.

Given these clinical needs, particularly for the incurable, aggressive, and rare entities of γδ T-NHL and NKTCLs, we aimed to design an HDAC6 inhibitor with drug-like properties, explored here via medicinal chemistry, X-ray crystallography studies, pharmacological and biochemical methodologies. NN-429 is an improved analog of older generation HDAC6 inhibitor molecules KT-531 and NN-390, with higher in vitro selectivity, target potency and in vivo half-life [10,27]. We specifically studied our novel HDAC6 inhibitor, NN-429, in a panel of human PTCL cell lines to explore targeted drug efficacy, finding the highest sensitives in hepatosplenic γδ T-NHL and NKTCL derived cell lines. Furthermore, we pioneered synergy screening with established drugs such as cytarabine, doxorubicin, etoposide, and SNS-032, which revealed that HDAC6 inhibition combined with chemotherapeutic drugs can deliver a synergistic benefit in γδ T-NHL or NKTCL.

## 2. Results

### 2.1. X-ray Crystallography Study of Precursor Molecule NN-390

**Figure 1:**
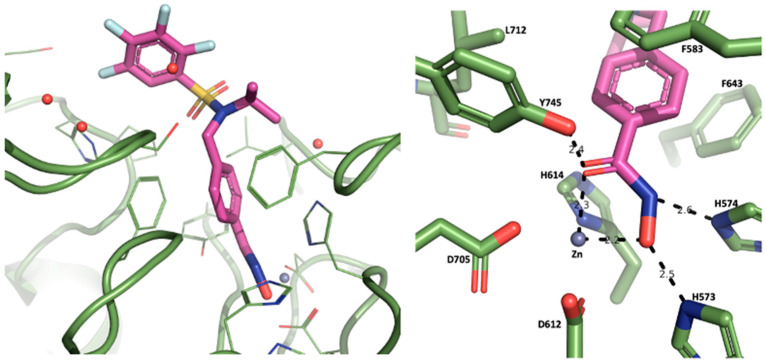
Crystal structure of NN-390 in the active site of HDAC6 surrounded by side chains of interacting residues. A close-up of the catalytic pocket shows the interaction mode of the hydroxamate moiety with the Zn^2+^ ion (blue-gray sphere) via a bidentate metal coordination (dashed black lines) and the surrounding residue side chains.

The X-ray crystal structure of the HDAC6–NN-390 complex, which was solved at 1.6 Å resolution (Figure 1), displays a similar profile of contacts in the catalytic tunnel when compared to that of HDAC6-ricolinostat complex. Unlike that of the HDAC6-TO-317 complex, the hydroxamate moiety coordinates to the catalytic Zn^2+^ ion in a bidentate fashion [28]. The hydroxamate N-O group coordinates to Zn^2+^ with an average separation of 2.2 Å and the hydroxamate C=O group with a distance of 2.34 Å. Alongside these contacts, interaction of NN-390 completes the canonical 5-membered chelate complex via a hydrogen bond network. The chelate complex involves the side chains Y745 (O—O distance = 2.44 Å), H574 (N—N distance = 2.62 Å), and H573 (N—O distance = 2.47 Å), the latter of which is unobserved for TO-317. Furthermore, all of these polar interactions are shorter and closer in contact compared to those of ricolinostat. The hydroxamate moiety of NN-390 is further able to engage in hydrophobic interactions with D612, D705, and H614, similar to that of ricolinostat. The H614 side chain makes Van der Waals contacts with the benzyl group of the phenylhydroxamate moiety and the isopropyl side group in NN-390, the latter of which is mediated via a water molecule. The benzyl group is sandwiched between F583 and F643 and establishes π-π stacking interactions. The isopropyl side group is closely situated with the F643 side chain and engages in hydrophobic interactions. The F583 side chain alongside L712 chain also engages in Van der Waals contacts with one of the oxygen atoms of the sulfonamide moiety. Although relatively weakly resolved, and likely more dynamic, the tetra-fluorobenzene (TFB) capping group is observed to make weak interactions with the side chain of H463 and that of S531 and F583 via distinct water molecules.

### 2.2. Chemistry

**Scheme 1:**
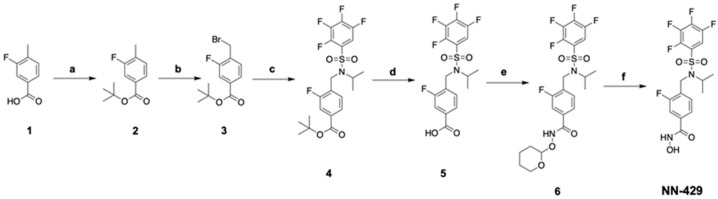
Reagents and conditions to synthesize NN-429. (**a**) ^t^BuOH, EDC, DMAP, THF, 18 h, RT; (**b**) NBS, AIBN, CCl_4_, 4–6 h, 90 °C; (**c**) 2,3,4,5-tetrafluoro-*N*-isopropylbenzenesulfonamide, Cs_2_CO_3_, ACN, 6–18 h, RT; (**d**) 4 M HCl/dioxane, 3–16 h, RT; (**e**) (i) (COCl)_2_, THF, DMF, 1 h, 0 °C; (ii) H_2_N-OTHP, ^i^Pr_2_NEt, THF, 16 h, RT; (**f**) 4 M HCl/dioxane, 3–16 h, RT.

The synthetic pathway used to furnish NN-429 is outlined in Scheme 1. Synthesis of NN-390 has been previously described [27]. *Tert*-butyl ester protection of 3-fluoro-4-methylbenzoic acid (**1**) led to the generation of tert-butyl 3-fluoro-4-methylbenzoate (**2**, 70%), which was then brominated at the benzylic position (**3**, 56%). S_N_2 reaction of 2,3,4,5-tetrafluoro-*N*-isopropylbenzenesulfonamide and the brominated product formed tert-butyl 3-fluoro-4-(((2,3,4,5-tetrafluoro-*N*-isopropylphenyl)sulfonamido)methyl)benzoate (**4**, 76%). Acid-mediated hydrolysis of the carboxylate ester generated the free carboxylic acid product (**5**, 92%), which coupled to tetrahydropyranyl (THP) (**6**, 71%). A final deprotection using 4 M HCl in dioxane resulted in NN-429, which was purified using preparative HPLC (44%).

### 2.3. Biochemical and Biophysical Characterization of HDAC6-Selective Inhibitor NN-429

**Figure 2:**
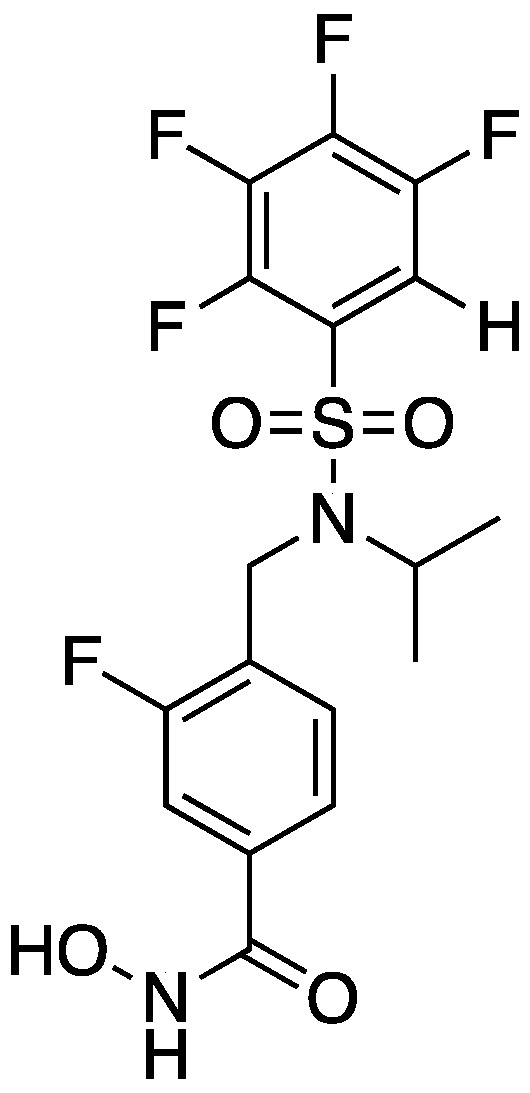
Structure of NN-429. Synthesis of NN-429 available in Supporting Information Scheme S1.

**Figure 3:**
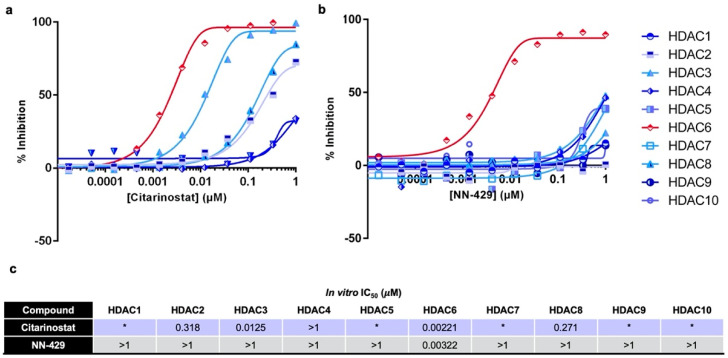
(**a**). Dose–response curves representing the percent inhibition of citarinostat against HDAC2, HDAC3, HDAC4, HDAC6 and HDAC8 via EMSA. (**b**). Dose–response curves representing the percent inhibition of NN-429 against HDAC1-10 via EMSA (Nanosyn, CA, USA). (**c**). In vitro HDAC inhibition IC_50_ values of citarinostat and NN-429 against HDAC1-10.

Following the discovery and preclinical evaluation of HDAC6-selective inhibitor NN-390, efforts were focused on optimizing its cellular behavior and ADME/PK profile [27]. A single point change via the addition of a fluorine atom meta to the hydroxamic acid of the scaffold, led to the generation of NN-429 (Figure 2). With a stronger in vitro potency towards HDAC6 (IC_50_ = 3.2 nM; 3-fold more potent than NN-390), and in vitro HDAC6 selectivity of at least 312-fold over all HDAC isoforms, NN-429 was an evidently more attractive inhibitor than precursors KT-531 and NN-390 (Figure 3) [10,27]. In the absence of a crystal structure of NN-429 due to poor crystal formation, we anticipate NN-429 to adopt a similar conformation as its precursor molecule NN-390 (Section 2.1).

Given such encouraging target potency and a striking preference for HDAC6, further biochemical and biophysical evaluations of NN-429 were conducted. To probe the selectivity across the whole family of HDAC proteins, a functional inhibitory selectivity screen (EMSA, Nanosyn, CA, USA) was conducted against 10 isoforms as well as a comparison to citarinostat (Figure 3a–c). Citarinostat demonstrated modest selectivity for the target isoform (HDAC6), with obvious inhibition of HDAC2, HDAC3, and HDAC8 as well (Figure 3a). In contrast, NN-429 showed no significant activity towards any of the other HDAC isoforms up to 1 µM (highest concentration evaluated) (Figure 3b,c). NN-429 has a commendable in vitro HDAC6 selectivity of >312-fold across the HDAC family.

**Figure 4:**
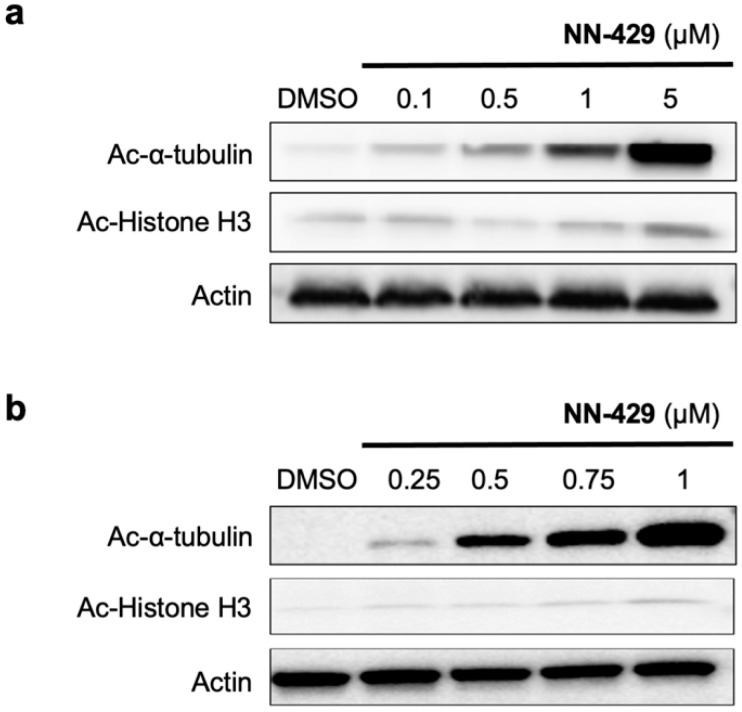
(**a**). Western blot illustrating α-tubulin acetylation and histone H3 acetylation levels in the (**a**) multiple myeloma (MM.1S) and (**b**) acute myeloid leukemia (MV4-11) cell line following 6 h treatment with varying concentrations of NN-429. Protein extracts were prepared, resolved by SDS-PAGE and immunoblotted with acetylated α-tubulin (Ac-α-tubulin), acetylated histone H3 (Ac-Histone H3) and actin antibodies. Representative Western blots of three independent experiments are shown.

In cellulo target engagement was assessed via Western blotting of HDAC6 substrate α-tubulin, and HDAC Class I substrate histone H3 in multiple myeloma (MM.1S) cells and acute myeloid leukemia (AML) cells (MV4-11; Figure 4). A dose-dependent increase in the acetylation of α-tubulin was observed at as low as 0.1 µM of NN-429, with visible off-target interaction only at 5 µM in MM.1S cells (Figure 4a). In MV4-11 cells, strong target engagement with minimal acetylation of off-target histone H3 was observed for NN-429-treated cells (Figure 4b).

**Figure 5:**
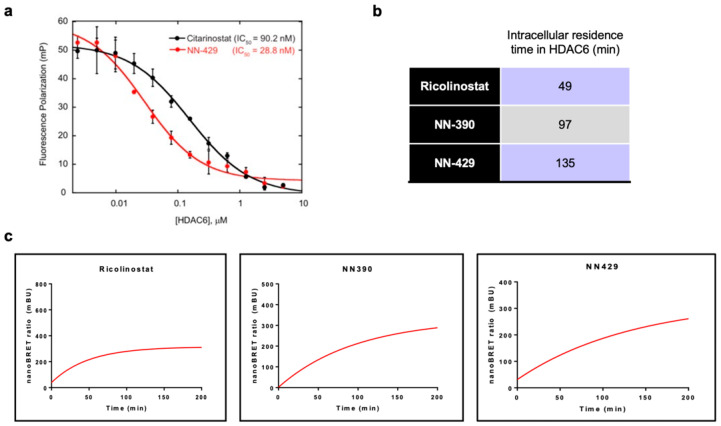
(**a**). Dose–response curves of NN-429 and citarinostat against recombinant *zf*HDAC6 using fluorescence polarization. Fluorescence polarization IC_50_ data against *zf*HDAC6 is provided. (**b**). In cellulo residence time of selected compounds for HDAC6 target engagement in HeLa cells transiently expressing HDAC6-NanoLuc full-length fusion protein. (**c**). Residence time analysis of ricolinostat, NN-390 and NN-429 in HDAC6 within live cells.

A fluorescence polarization (FP) assay was employed as an orthogonal biochemical assay to validate EMSA activity findings. NN-429 displayed an IC_50_ of 28.8 nM, while citarinostat had a 3-fold lower binding affinity (IC_50_ of 90.2 nM) (Figure 5a). Although the inhibitory activities towards HDAC6, through the EMSA assay, were similar for these two compounds (IC_50 NN-429_ = 3.22 nM, IC_50 Citarinostat_ = 2.21 nM) (Figure 3), the FP assay revealed a divergence in binding affinity.

**Figure 6:**
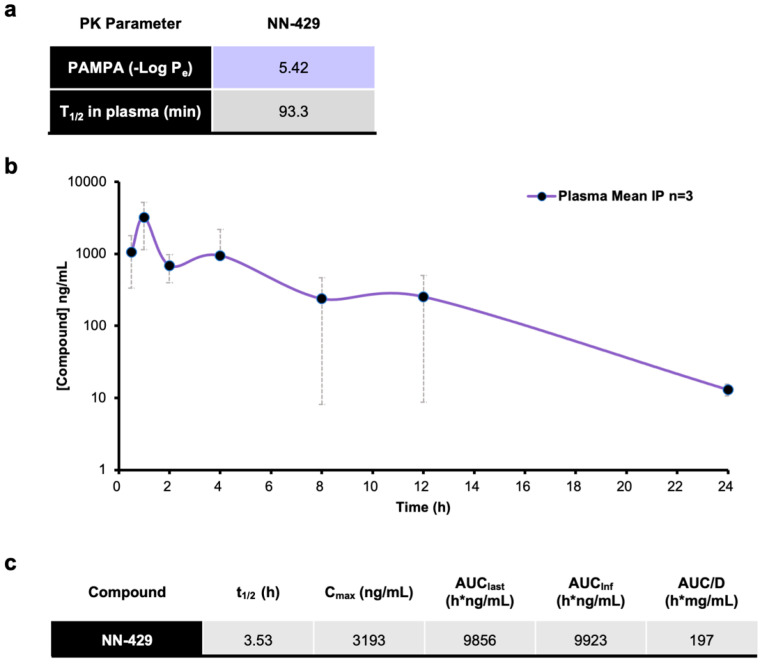
(**a**). PAMPA and plasma stability evaluation of NN-429. (**b**). In vivo PK profile of NN-429 (IP; 50 mg/kg), in male CD-1 mice (*n* = 3). (**c**). In vivo PK parameters of NN-429 (IP; 50 mg/kg), in male CD-1 mice (*n* = 3).

To obtain a more biologically relevant intracellular binding measurement, NN-429 was assessed for HDAC6 residence time in HeLa cells via a NanoBRET™ assay [29,30]. In contrast to the previous generation inhibitor, NN-390, NN-429 exhibited a longer residence time of 135 min (NN-390-HDAC6 residence time = 97 min) (Figure 5b,c) [27]. The increased residence time improves the longevity of the HDAC6 inhibitory effect. In vitro and in vivo ADME/PK analyses were conducted to assess the pre-clinical tractability of NN-429 (Figure 6). Cell permeability was explored via a PAMPA experiment, where NN-429 possessed good permeability with a permeability coefficient (−Log Pe) of 5.42 (−LogPe < 6 is considered highly permeable) (Figure 6a). This was in line with Western blot findings, where strong target engagement was observed at sub-micromolar concentrations, indicating that cell permeability was not a limiting factor for the in cellulo drug-like profile of NN-429.

We also investigated the in vivo pharmacokinetics of NN-429 in male CD-1 mice (*n* = 3) (intraperitoneal (IP); 50 mg/kg), to establish parameters such as t_1/2_, C_max_ and AUC_last_ (Figure 6b,c). NN-429 exhibited a half-life of 3.53 h in CD-1 mice, with C_max_ and AUC_last_ of 3193 ng/mL and 9856 h*ng/mL, respectively. This was a notable improvement from the PK profiles of predecessors KT-531 and NN-390, although the dose administered to the mice was higher in the case of NN-429. Nevertheless, in vivo t_1/2_ is generally not impacted by dosage changes, and the t_1/2_ of NN-429 was >3.5-fold longer than that of KT-531, and ~1.8-fold longer than the t_1/2_ of NN-390 [10,27]. In essence, NN-429 demonstrated better in vivo exposure, and demanded further attention as an exciting pre-clinical lead. The extended HDAC6 residence time and improved PK profiles makes NN-429 a much more attractive preclinical candidate for HDAC6-reliant diseases.

The previous analog NN-390 had displayed efficacy in models of brain cancer, most significantly Group 3 Medulloblastoma [27]. However, NN-429 displayed extremely poor exposure in the brain of CD-1 mice. Via IP dosing of 50 mg/kg in male CD-1 mice (*n* = 3), there were no observable levels of NN-429 in the brain, implying that this molecule fails to cross the blood–brain barrier (BBB) (see Supporting Information Figure S1). In this context, NN-429 was explored in hematological indications.

### 2.4. NN-429 Displays Efficacy in NKTCL

#### 2.4.1. Selective Cytotoxicity of NN-429 in Peripheral T-Cell Lymphomas (PTCL)

**Figure 7:**
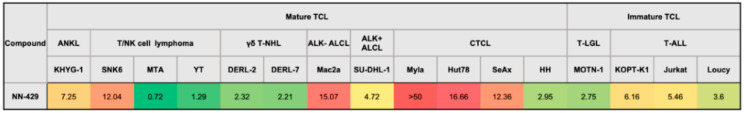
NN-429 shows selective cellular cytotoxicity in a spectrum of TCL malignancies. A heatmap of IC_50_ values (µM) is shown, calculated from drug response analysis of NN-429 from one representative out of three independent experiments. Cell lines are classified according to the respective T-cell leukemia/lymphoma entity. Abbreviations: ANKL, aggressive NK-leukemia; NK/T, Natural killer/T-cell; γδ T-NHL, γδ T-cell non-Hodgkin lymphoma; ALK−ALCL, anaplastic large cell lymphoma (anaplastic lymphoma kinase negative); ALK+ALCL, anaplastic large cell lymphoma (anaplastic lymphoma kinase positive); CTCL, cutaneous T-cell lymphoma; T-LGL, T-cell large granular lymphocytic leukemia; T-ALL, T-cell acute lymphoblastic leukemia. IC_50_ values (µM) calculated from drug response analysis of NN-390, KT-531 and citarinostat available in Supporting Information Table S1.

A wide collection of mature and immature T-cell lymphoma (TCL) cell lines encompassing eight distinct T cell cancer groups, comprising a total of sixteen authenticated and mycoplasma-free cell lines derived from individual patients, were analyzed to assess cytotoxic death induction of NN-429 (Figure 7).

NN-429 displayed a unique selective cytotoxicity profile in TCL (Figure 7). Particularly impressive cytotoxicity was observed for lines derived from the mature TCL malignancies of γδ T-NHL and NKTCL, while lines from other mature TCL such as cutaneous TCL (CTCL) and anaplastic large cell lymphomas (ALCLs) displayed unresponsiveness to the inhibitor (Figure 7). In contrast to the mature TCL, immature TCL cell models such as Jurkat and KOPT-K1 displayed moderate sensitivity to the highly selective HDAC6 inhibitor. In the HSTL derived cell line DERL-2, a dose-dependent increase in cells undergoing apoptosis was observed with NN-429 (Supporting Information Figure S2). Flow-cytometry based, annexin V/PI staining revealed that 15% of cells were undergoing early-stage apoptosis (annexin V+/PI−) after 18 h treatment with 0.1 μM of NN-429, and 56% of cells were in early-stage apoptosis following a 5 μM treatment.

#### 2.4.2. Combination Studies of NN-429 with Clinical Agents in γδ T-NHL and NKTCL

To avoid and overcome resistance, a significant challenge in PTCL, novel drug combinations of components that target more than one cellular pathway represent a key strategy to be investigated more intensely in these tumors [31,32,33]. Furthermore, combination treatments have allowed alleviation of drug-induced toxicity and adverse effects, by enabling administration of each drug at lower doses [32,34]. Here, we explored the rational design of pairwise drug combination evaluation via a range of functional screening assays.

**Figure 8:**
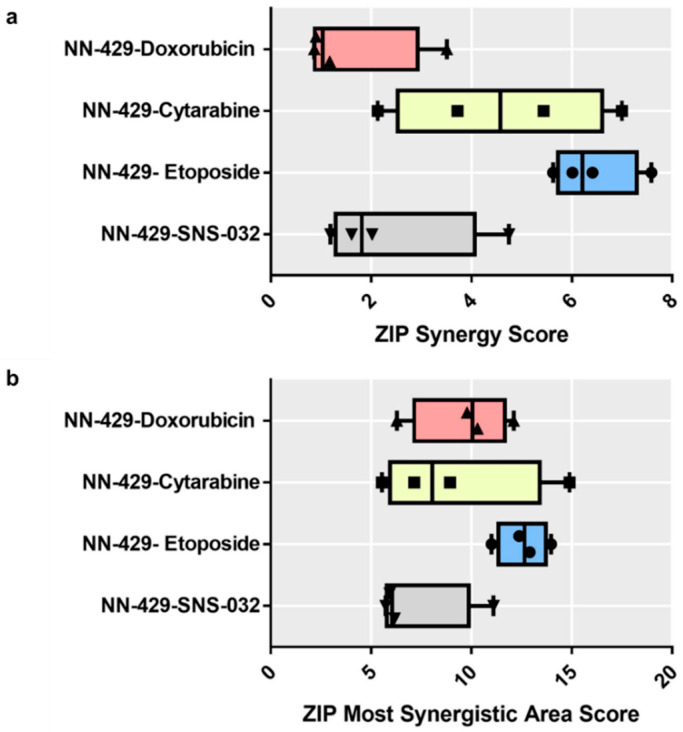

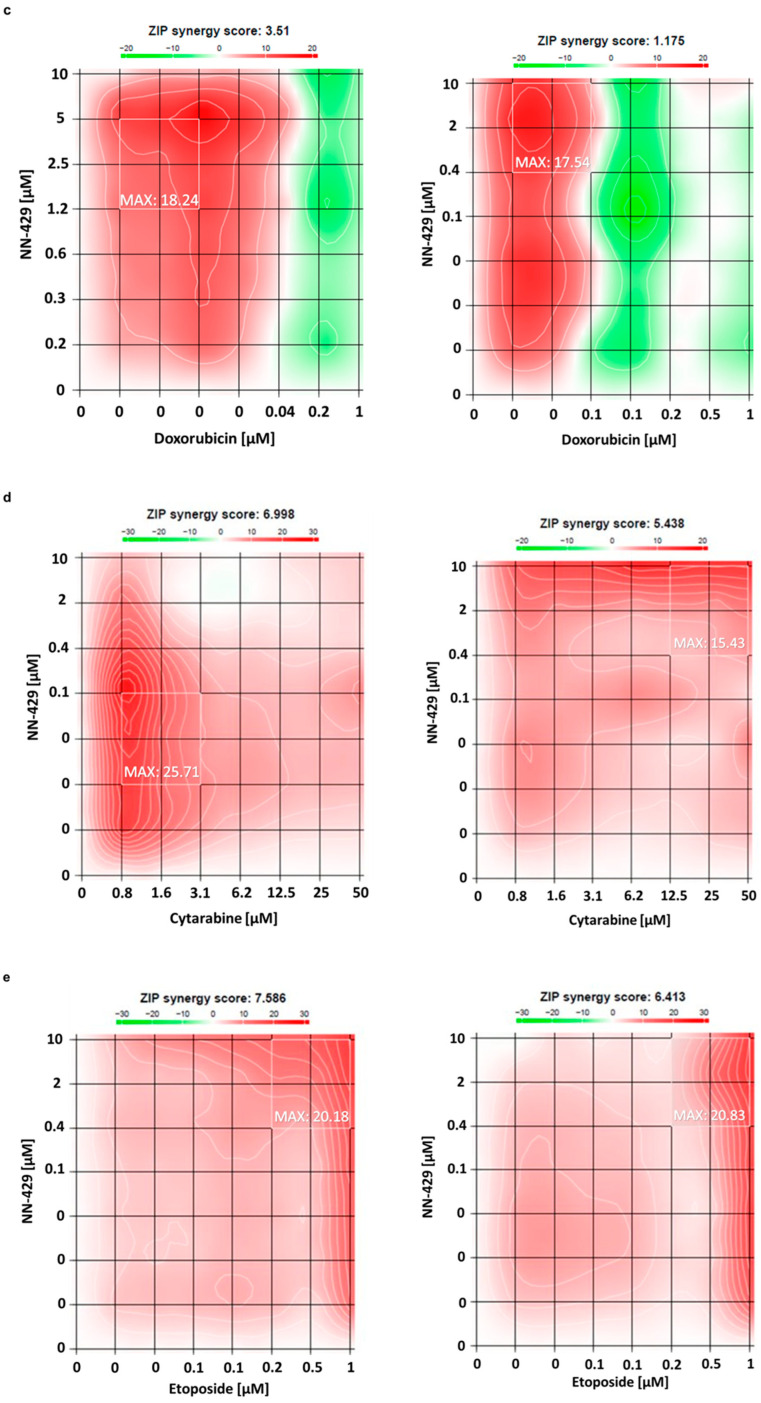

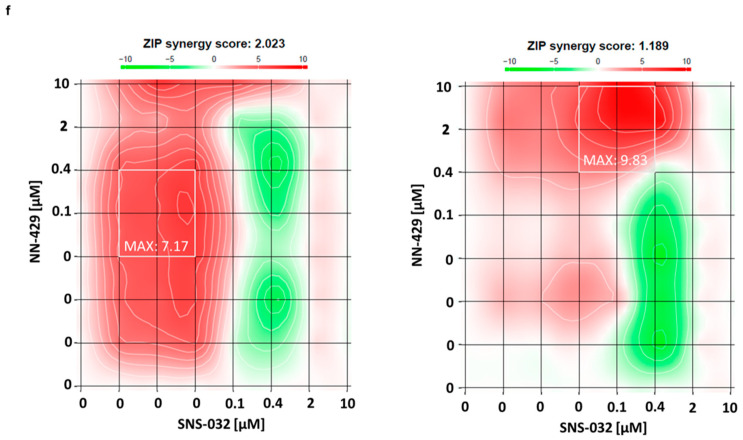
Synergy of NN-429 with clinical agents cytarabine, doxorubicin, etoposide, and with SNS-032 in YT cells. (**a**) Box plot of ZIP synergy scores of NN-429 with doxorubicin, cytarabine etoposide, and SNS-032 in YT cells. (**b**) Box plot of MSA scores of NN-429 with doxorubicin, cytarabine, etoposide and SNS-032 in YT cells. Two representative interaction landscapes between (**c**) NN-429 and doxorubicin, (**d**) NN-429 and cytarabine, (**e**) NN-429 and etoposide, and (**f**) NN-429 and SNS-032 in YT cells. The MSA is outlined by a white square and the MSA score is indicated in the square.

Cytarabine, doxorubicin, and etoposide are standard chemotherapeutic agents that are components of widely used regimens in PTCL, including γδ T-NHL and NKTCLs. Following our findings of selective cellular cytotoxicity in the NK lymphoma cell line YT (Figure 8), NN-429 was individually combined with each of these drugs and tested on YT cells to investigate synergistic interactions (Figure 8). To analyze the efficacy of drug combinations, the web application SynergyFinder was utilized [35,36]. The zero interaction potency (ZIP) synergy model designed by Yadav et al. was employed to capture the drug interaction relationships, which compares the change in potency of the dose–response curves between mono-agent drugs versus their combinations [37]. ZIP scores were obtained from the analysis of dose–response matrix experiments, where two agents were tested at various dose pairs in a serially diluted manner. A positive ZIP score indicated a synergistic relationship, while a negative value suggested an antagonistic interaction. Each drug combination consists of an overall ZIP synergy score which is the score for all tested concentrations. Additionally, a most synergistic area (MSA) score is also provided representing the ZIP score for a 3-by-3 concentration range with the highest ZIP.

The NN-429-etoposide combination exhibited the strongest synergism with the highest average most synergistic area score (>10 indicates synergy) (Figure 8b). Combination treatment of NN-429-etoposide had an average overall ZIP synergy score of 6.41 which suggests an overall additive effect (Figure 8a and Supporting Information Figure S3). However, the average most synergistic area score of 12.57 (Figure 8a,c) suggests high synergy in the area from 0.2 µM to 1 µM of etoposide and 0.4 µM and 10 µM of NN-429 (highlighted with the white square in (Figure 8e). The combination of NN-429 with cytarabine resulted in an average overall ZIP synergy score of 4.57, with the most synergistic area score of 9.13, both ZIP scores stating an additive effect of the drug combination (Figure 8a,b and Supporting Information Figure S3). The anthracycline doxorubicin demonstrated in combination with NN-429 a lower overall ZIP synergy score of 1.61, and a most synergistic area score of 9.62 also suggesting an overall additive effect (Figure 8a,b and Supporting Information Figure S3)**.** The most synergistic area for this combination was in the low nanomolar range for doxorubicin which is ideal for clinical translation (from 0.06 nM to 1.6 nM, Figure 8c). Finally, combination of NN-429 with the CDK2/7/9 inhibitor SNS-032 showed a similar effect to doxorubicin with an average overall ZIP synergy score of 2.39 and the most synergistic area score of 7.23 also suggesting an additive effect (Figure 8a,b and Supporting Information Figure S3). Similar to doxorubicin, the most synergistic area for the NN-429-SNS-032 combination was in the low nanomolar range for SNS-032 (Figure 8f). This data could be of preclinical significance, given the ability of these novel and distinctive drug target combinations to pave new therapeutic avenues.

**Figure 9:**
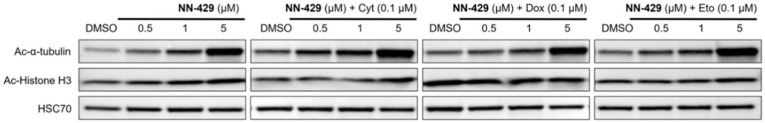
Western blot illustrating α-tubulin acetylation and histone H3 acetylation levels in YT cells following 24 h treatment with varying concentrations of NN-429, NN-429 + 0.1 µM cytarabine (cyt), NN-429 + 0.1 µM doxorubicin (dox), NN-429 + 0.1 µM etoposide (eto). Protein extracts were prepared, resolved by SDS-PAGE, and immunoblotted with acetylated α-tubulin, acetylated Histone H3 and HSC70 antibodies were used for loading controls. A representative Western blot of three independent experiments is shown.

Next, the impact on HDAC6 inhibition and subsequent acetylation increase in HDAC6 substrates was explored following combination treatments. Western blot of NN-429 in YT and DERL-7 cells showed a similar profile as NN-429 in AML (MV4-11) cells and multiple myeloma (MM.1S) cells (Figure 4 and Figure 9, and Supporting Information Figure S4). Western blotting with varying concentrations of NN-429, NN-429 + 0.1 µM cytarabine, NN-429 + 0.1 µM doxorubicin and NN-429 + 0.1 µM etoposide were conducted in YT and DERL-7 cells (Figure 9 and Supporting Information Figure S4, respectively). No observable changes in the mechanism of inhibition were imposed upon drug combinations in any of the cell lines, by any of the combinatory agents. As such, there was no significant change in the acetylation of HDAC6 substrate α-tubulin, and HDAC Class I substrate histone H3 following the addition of cytarabine, doxorubicin, etoposide and SNS-032 (see Supporting Information Figure S5). This is consistent with the established mechanisms of action of the clinical agents, which portray no overlapping pathways with HDAC6 function.

## 3. Discussion

Throughout the last fifteen years, targeting the classical HDAC family and more recently, individual HDAC isozymes, has been found to be effective for various therapeutic purposes. Clinically approved HDAC inhibitors include SAHA, romidepsin, belinostat, and panobinostat by Novartis [38,39,40,41]. These highly potent and clinically efficacious drugs function as pan-HDACi, exhibiting broad spectrum anti-HDAC activity and targeting multiple isozymes with comparable affinities.

The clinical utility of these pan-HDACi has been limited due to the toxicities they impose. Adverse effects such as fatigue, diarrhea, vomiting, anorexia, asthenia, weight loss, and thrombocytopenia are often reported due to the non-specific targeting of current HDACi drugs [42]. Despite being in the same classical HDAC family, the individual HDAC proteins vary substantially in their function, and the inhibition of more than one family member may lead to undesirable effects [43]. The pleiotropic nature of the cellular response of pan-HDAC inhibition reflects a global disruption in epigenetic and cytoplasmic processes that are hard to decipher. However, the effects of these epigenetic modulators in cells is a heavily complicated question, although the development of selective HDAC inhibitors and probes has shed some light on the roles of each isozyme in health and disease. There is burgeoning interest in the development of isozyme-selective HDAC inhibitors, and the past two decades have witnessed almost a thousand clinical trials in the HDAC area since the discovery of SAHA [44,45,46,47]. Mice lacking HDAC6 have a benign phenotype with no detrimental impact on viability, fertility, or lymphoid development, suggesting that selective HDAC6 inhibition is a safe therapeutic strategy [48].

Several moderate-to-highly selective HDAC6 inhibitors have emerged, and led to clinical attempts of HDAC6 intervention with drug candidates ricolinostat (ACY-1215), citarinostat (ACY-241), KA2507, CKD-504, CKD-506, and CKD-510 [44,46,49,50,51,52]. Citarinostat is undergoing clinical evaluation in a range of neoplasms, notably multiple myeloma, malignant melanoma, non-small cell lung cancer (NSCLC) and other solid tumors including those of the reproductive tract or the GI tract, mesothelioma or head and neck cancers [45,50].

The newer generation HDAC6 candidate KA2507 aims to tackle the selectivity constraints of ricolinostat and citarinostat, with superior activity in in vitro biochemical assays that translates into confirmed selectivity in patients [44]. However, poor oral bioavailability and unsatisfactory ADME of KA2507 necessitates ongoing work to effectively optimize these crucial drug parameters for a well-rounded HDAC6-targeting clinical candidate.

This presented study represents novel exploratory findings of HDAC6 inhibition in γδ T-NHL and NKTCL, and its combination with the clinical agents cytarabine, doxorubicin, etoposide and SNS-032. NN-429, an HDAC6-selective inhibitor with in vitro HDAC6 selectivity of >312-fold across other HDAC isoforms, and HDAC6 potency of IC_50_ of 3.2 nM, is an improved analog of NN-390. This new generation inhibitor demonstrated a sustained duration of action against HDAC6, with an in cellulo residence time of 135 min, in contrast to 97 min for NN-390. Excitingly, NN-429 had a commendable half-life in CD-1 mice (t_1/2_ = 3.53 h), with a C_max_ of 3193 ng/mL and AUC_last_ of 9856 h*ng/mL. With limited brain bioavailability, NN-429 was deemed suitable for hematologic indications.

Unique selective cytotoxicity towards γδ T-NHL and NKTCL cell line systems was observed from exploring eight different T-cell cancer types and a total of sixteen different patient-derived cell line models. Furthermore, we carried out synergy screens involving NN-429 and routinely used chemotherapeutic agents in these PTCL lines. Drug combinations were explored, particularly with cytarabine, doxorubicin, etoposide and SNS-032, by implementing a ZIP scoring method via SynergyFinder. The overall synergy score was calculated as a deviation of phenotypic response compared to expected values, over the full dose–response matrix of the two drug combinations. The NN-429-etoposide combination exhibited the strongest synergy observed in YT cells, with an average most synergistic area score of 12.57 (>10 is synergistic). The combination of NN-429 with doxorubicin, cytarabine, and SNS-032 illustrated an overall additive effect with overall synergy scores between 1 and 10, although clear areas of synergies could be further explored, especially around the dose regions of the maximum synergy score. Given the many negative side effects of chemotherapeutics, e.g., vascular damage, secondary malignancies, infertility, future studies might explore other drug categories to be combined with HDAC6 inhibitors, such as tyrosine kinase inhibitors or other epigenetic blockers. However, chemotherapeutic combination therapy with a targeted agent such as our HDAC6 selective blocker is generally more rapidly translatable. Furthermore, considering cancer treatment costs, this strategy would also be comparably cheaper than targeted therapy combinations, which were considerations for us to go into more comprehensive testing.

In summary, the highly selective HDAC6 inhibitor NN-429 exhibited strong selective cytotoxicity as a single agent, and synergy with clinical agents cytarabine, doxorubicin, etoposide, and SNS-32 in cellular models of γδ T-NHL and NKTCL. Although the HDAC6 substrate and off-target Western blots have confirmed no drug–drug interactions or overlap in mechanism, similar studies must be replicated for the biological pathways of cytarabine, doxorubicin and etoposide. Selective HDAC6 inhibitors may therefore have clinical utility in γδ T-NHL and NKTCL, and this warrants further investigations towards clinical translation. Our selective HDAC6 inhibitor can be further improved with medicinal chemistry efforts and future trials might be possible with a top lead compound to be explored in rare T- or NK cell leukemias/lymphomas in line with the clinical need to find less toxic and more specific drugs.

## 4. Materials and Methods

### 4.1. Protein Expression

The gene (NCBI: XP_009302026.1) corresponding to HDAC6 from Danio rerio (zebrafish; catalytic domain 2, S440-R798) was codon-optimized, synthesized, and cloned into a pET-28b(+) vector using restriction enzymes *Nhe*I and *Xho*I with a N-terminal His-SUMO tag. Molecular cloning was performed by GenScript. BL21 (DE3) RILP cells were transformed with the generated plasmid (containing His-SUMO-HDAC6) and single colonies were selected and cultured in 5 mL of Super broth containing kanamycin (50 µg·mL^−1^) and chloramphenicol (34 µg·mL^−1^). The cultures were grown with continuous shaking at 37 °C for 4 h and used to inoculate 1 L of Super broth containing 10 mM MgSO_4,_ 0.1% (*w*/*v*) glucose, kanamycin (50 µg·mL^−1^) and chloramphenicol (34 µg·mL^−1^). Following culture growth (OD_600_ = 2.0), the incubation temperature was reduced to 18 °C, and the media was supplemented with 0.5 mM zinc chloride solution, 3% (*v*/*v*) ethanol, and 0.5 mM IPTG. The cells were harvested after 18–20 h and stored at −80 °C.

### 4.2. Protein Purification and Crystallization

HDAC6 cell pellets were lysed via sonication in 20 mM Tris-HCl pH 8.0, 100 mM arginine, 100 mM glutamic acid, 5 mM β-mercaptoethanol, 5 mM imidazole, 0.2% [*v*/*v*] Triton-X, 0.1% [*v*/*v*] Nonidet P-40 substitute, 10% [*v*/*v*] glycerol, 2 mg/mL deoxycholic acid, 1 mg/mL lysozyme, 5 mM 6-aminocaproic acid, 5 mM benzamide and 1 mM phenylmethylsulfonyl fluoride). The cell lysate was centrifuged at 14,800× *g* for 30 min to remove insoluble particles and the supernatant was filtered and loaded onto a 5 mL Ni^2+^-nitrilotriacetic acid (NTA) affinity column pre-equilibrated with 20 mM Tris-HCl pH 8.0, 150 mM NaCl, 5 mM imidazole, 10% [*v*/*v*] glycerol, 5 mM β-mercaptoethanol. The lysate was passed through the column by gravity and washed with 10 CV of wash buffer 1 (20 mM Tris-HCl pH 7.4, 500 mM NaCl, 5 mM imidazole, 10% [*v*/*v*] glycerol, 5 mM β-mercaptoethanol), followed by 5 CV of wash buffer 2 (20 mM Tris-HCl pH 7.4, 500 mM NaCl, 45 mM imidazole, 10% [*v*/*v*] glycerol, 5 mM β-mercaptoethanol). The His-SUMO-HDAC6 protein was eluted from the nickel column using elution buffer (20 mM Tris-HCl pH 7.4, 150 mM NaCl, 500 mM imidazole, 10% [*v*/*v*] glycerol, 5 mM β-mercaptoethanol). The eluted fractions containing His-SUMO-HDAC6 protein were diluted two-fold with dilution buffer (20 mM Tris-HCl pH 7.4, 150 mM NaCl, 10% [*v*/*v*] glycerol, 5 mM β-mercaptoethanol) and was cleaved with His-Ulp1 protease. The cleaved protein was concentrated using a 10 kDa cutoff centrifugal concentrator, and further purified with a gel filtration column in 50 mM HEPES pH 7.5, 100 mM KCl, 5% glycerol (*v*/*v*), 0.1 mM TCEP and immediately supplemented with final 5 µM compound (50% DMSO stock). Fractions were pooled, (1 mM TCEP was added) and concentrated to 10–15 mg/mL and further mixed with 1 mM compound. Samples were incubated at 4 °C for 1 h and plated onto crystallization plates.

Crystals of compound bound HDAC6 protein were grown for 7–10 days in 0.2 M potassium thiocyanate, 12% (*w*/*v*) PEG3350 at 4 °C. Crystals were harvested from the drops, briefly soaked in 25% ethylene glycol and stored in liquid nitrogen.

### 4.3. Data Collection with Structure Solution and Refinement

X-ray diffraction data for HDAC6 was collected on NE-CAT beamline 24-ID-C at the Advanced Photon Source; data was collected on a Pilatus 6M detector with 0.2 s exposure and 0.2° oscillation per frame (λ = 0.979 Å). Diffraction images were processed using the Xia2 [53] and the structure was solved by molecular replacement with Phaser-MR [54,55,56,57,58] using 6CSR (PDB) as the search model. The structures were refined within Phenix [59], with manual examination/rebuilding of |2*F*_o_| − |*F*_c_| and |*F*_o_| − |*F*_c_| maps using Coot [58]. Stereochemical quality of the final refined structures was analyzed via MolProbity [60], and deposited in the PDB as 7UK2 with the corresponding statistics provided in Supplementary Table S2. Structures were visualized through Pymol.

### 4.4. HDAC Target Engagement (Nanosyn, CA, USA)

In vitro HDAC inhibition assays (EMSA) were carried out by Nanosyn using a microfluidic electrophoresis instrument (Caliper LabChip^®^ 3000, Caliper Life Sciences/Perkin Elmer) which was used to detect the concentrations of both de-acetylated and acetylated FAM-labelled peptide substrates following an activity-based assay. The deacetylation of the peptide substrates alters the electrophoretic mobility. HDAC proteins were pre-diluted in the assay buffer (100 mM HEPES, pH 7.5, 0.1% BSA, 0.01% Triton X-100, 25 mM KCl) and 10 μL of HDAC protein was added per well to a 384-well plate. Compounds were serially pre-diluted in DMSO and introduced to the HDAC protein samples using Labcyte Echo acoustic dispensing system, and the DMSO concentration was adjusted to 1% (*v*/*v*) in the protein-compound mixture. TSA, JNJ-26481585, and MS-275 were used as positive controls, whereas the absence of inhibitor (DMSO only) and the absence of enzyme were used as the negative controls representing 0% and 100% inhibition, respectively. Addition of 10 µL of the FAM-labelled substrate allows the reaction to start, which is followed by an incubation period. A change in the relative intensity of the acetylated peptide substrate and deacetylated product is used to determine the activity (product to sum ratio, PSR) using the following equation:

(PSR): P/(S + P)

where P is the peak height of the product, and S is the peak height of the substrate.

Percent inhibition (P_inh_) was determined as follows:

P_inh_ = (PSR_0%inh_ − PSR_compound_)/(PSR_0%inh_ − PSR_100%inh_) × 100

where PSR_compound_, PSR_0%inh_, and PSR_100%inh_ are the product to sum ratios in the presence of inhibitor, absence of inhibitor and absence of enzyme, respectively.

The IC_50_ values of all inhibitors were calculated by plotting compound concentration versus P_inh_ fitted to a 4-parameter sigmoid dose–response model on Xlfit software (IDBS).

### 4.5. Western Blotting

Cells (MV4-11 AML, MM.1S multiple myeloma, or YT cells) were incubated with inhibitors prior to washes (2×) with cold phosphate-buffered saline (PBS) and cell lysis with radioimmunoprecipitation assay (RIPA) buffer (20 mM Tris pH 7.4, 150 mM NaCl, 0.5% deoxycholate, 1% Triton X-100, and 0.1% sodium dodecyl sulfate (SDS)). Total protein content was determined through a bicinchoninic acid (BCA) assay (ThermoFisher Scientific, Waltham, MA, USA). The cell lysate proteins were separated via a 4–20% polyacrylamide SDS gel and transferred to a PVDF membrane (Bio-Rad, Hercules, CA, USA). Non-specific binding of the antibody to the membrane was reduced by blocking the membranes with a 5% (*w*/*v*) solution of Bovine Serum Albumin powder in TBS-T. This was followed by incubation at 4 °C (overnight) with the following antibodies: acetylated α-tubulin mouse monoclonal (sc-23950, Santa Cruz, Santa Cruz, CA, USA), acetylated Histone H3 (Ac-Lys18, 07-354, Sigma), β-Actin mouse monoclonal (AC-15, sc-69879, Santa Cruz), and Heat Shock Complex 70 HSC70 (sc-7298, Santa Cruz). Following overnight incubation, horseradish peroxidase (HRP)-conjugated goat anti-mouse IgG secondary antibody (7076, Cell Signaling) or HRP-linked anti-rabbit IgG secondary antibody (7074, Cell Signaling) was applied to the membrane in a 1:5000 dilution for 1 h. The bands were visualized using clarity Western ECL substrate luminal/enhancer solution and peroxide solution. Western blotting analysis was carried out using Image lab software (Bio-Rad).

### 4.6. Permeability Determination by PAMPA

1.8% solution (*w*/*v*) of lecithin in dodecane was added to each acceptor plate well (top), followed by application of the artificial membrane and addition of 300 μL of PBS (pH 7.4) solution to each well of the acceptor plate. Compounds were added to the donor plate and incubated at 25 °C, 60 rpm for 16 h. After incubation, aliquots of 50 μL from each acceptor well and donor plate were transferred into a 96-well plate, vortexed at 750 rpm for 100 s and centrifuged at 3220× *g* for 20 min. The concentration of the compounds was determined by LC-MS/MS.

The effective permeability (*P_e_*), in units of cm/s, was calculated using the following equation:

$$\log\;P_{e}=\log\;\{C\;\times \;[-\ln\;(1-\frac{\left[\mathrm{drug}\right]\mathrm{acceptor}}{\left[\mathrm{drug}\right]\mathrm{quilibrium}\;})]\}$$

where: C = VD × VA/[(VD + VA) × t × A]; VD = volume of donor compartment (0.30 mL); VA = volume of acceptor compartment (0.30 mL); A = filter area (0.24 cm^2^ for Multi-Screen Permeability Filter plate); and t = incubation time (in seconds).

### 4.7. Fluorescence Polarization (FP) Assay

The FP assay was conducted in a Greiner Bio-one black 384-well, nonbinding microplate (Cat 781900) as previous described [61,62]. These studies were performed in FP buffer (20 mM HEPES pH 8.0, 137 mM NaCl, 3 mM KCl, 1 mM TCEP, 5% DMSO). Binding experiments were performed in the presence of 50 nM FITC-M344 synthesized as described by Mazitschek et al. [63] and titrated with 0–3 µM HDAC6. Competition assays were performed by titrating 0–100 µM inhibitor to 300 nM HDAC6 CD2 and preincubating the samples for 10 min prior to addition of 50 nM FITC-M344 in FP buffer. The assay mixture was incubated for an additional 10 min before FP measurement. Polarization measurements were collected using Infinite M1000-Tecan (ex/em = 470 nm/530 nm) and data were plotted and fitted using Prism GraphPad 6 built-in function, log(inhibitor) vs. response—variable slope (four parameters).

### 4.8. Intracellular Target Engagement Residence Time Assay (nanoBRET)

NanoBRET target engagement intracellular HDAC assay was purchased from Promega (Cat.# N2080) and performed according to protocol. HeLa cells were grown in Dulbecco’s modified Eagle’s medium (DMEM) supplemented with 10% fetal bovine serum (FBS) (Sigma-Aldrich, St. Louis, MO, USA). In general, HeLa cells were cultivated, trypsinized, and resuspended to a density of 2 × 10^5^ cells/mL in assay medium (Opti-MEM I reduced serum media, no phenol red (Life Technologies Cat.# 11958-021)). To 20 mL the resuspended cells, 10 µg/mL of lipid complex consisting of 9:1 ratio of transfection carrier DNA to NanoLuc fusion DNA and 30 µL FuGENE HD transfection reagent (Promega, Cat.# E2311) in 1 mL assay medium was added. The cells were left to incubate overnight at 37 °C, 5% CO_2_ to generate a transient transfection containing NanoLuc-HDAC6 full length. The transiently transfected cells were treated with compound, and cells were centrifuged at 200× *g* for 5 min to pellet cells. Post incubation with substrate, the cell pellets were washed once with 1× PBS and dispensed on a white, nonbinding 96-well plate (Corning, Cat.# 3600) followed by 2× substrate + inhibitor solution and 20× tracer solution. The plate was shaken for 30 s at 750 rpm. Full occupancy control was performed in the absence of inhibitor and background control was performed in the absence of tracer (10 µL tracer dilution buffer only). NanoBRET measurements were collected using BioTek Cytation 3 (em = 450/50 nm, 610/LP nm, integration time = 1 s, delay = 100 ms) in 2 min interval. NanoBRET ratio was calculated using the equation below:

$$\mathrm{BRET}\;\mathrm{Ratio}\;(\mathrm{mBU})=(\frac{\mathrm{Acceptor}\;\mathrm{sample}}{\mathrm{Donor}\;\mathrm{sample}\;}\;-\frac{\mathrm{Acceptor}\;\mathrm{background}}{\mathrm{Donor}\;\mathrm{background}\;})\;\times \;1000$$

The BRET ratio was then plotted over time and fitted on Prism GraphPad 6 using the equations below to obtain residence time calculation:

Y = Y_0_ + (Plateau − Y_0_) × (1 − e−^kobs×t^)

t_1/2_ = 0.693 × residence time

### 4.9. FACS Apoptosis Detection Assay

DERL-2 cells were seeded, dosed with inhibitors, incubated for 18 h, and washed with ice cold 1× PBS. The resulting cell pellets were resuspended in 1× Binding Buffer (1 × 10^6^ cell/mL) from the FITC Annexin V Apoptosis Detection Kit I (BD Pharmingen). Subsequently, the dyes Annexin V (5 μL) and Propidium Iodide (PI, 5 μL) were added to 2.5 × 10^5^ cells (250 μL). The suspension was thoroughly mixed and incubated in the dark for 15 min. Following the addition of 250 μL of 1× binding buffer the cells were analyzed by flow cytometry within 1 h using Cytoflex S (Beckman Coulter, Brea, CA, USA).

### 4.10. In Vivo PK Study in CD-1 Male Mice (Pharmaron, MA, USA)

In vivo mouse studies were performed at Pharmaron in CD-1 male mice in triplicate. The test compounds were formulated within a 4 mg/mL solution (10% DMA, 65% PEG400, 25% saline). CD-1 mice were administered the test compound (50 mg/kg, I.P) once, and blood samples were obtained from each mouse at 0.25, 0.5, 1, 2, 4, 8, and 24 h post-dose. The mice had free access to food and water, were inspected for clinical signs and were weighted once prior to dosing. The working solutions of 5 μL at different concentrations (2, 4, 10, 20, 100, 200, 1000, 2000 ng/mL) were added to CD-1 mouse plasma (10 μL) to generate calibration standards of 1, 2, 5, 10, 50, 100, 500, and 1000 ng/mL. Four quality control (QC) samples at 2, 5, 50, and 800 ng/mL for plasma were prepared independently of calibration curves. Standards, QC samples, and unknown samples (total volume 15 µL) were added to acetonitrile (200 μL) containing IS (2 ng/mL Verapamil, and 50 ng/mL Dexamethasone) for precipitation of protein. Samples were vortexed and centrifuged (4 °C, 3900 rpm, 15 min), and the supernatant was diluted 3× with ultra-pure water. Diluted supernatant was injected into the LC-MS/MS system for quantitative analysis.

### 4.11. Cell Lines

The TCL cell lines, KHYG-1, SNK6, MTA, DERL-2, DERL-7, Mac2a, SU-DHL-1, Myla, Hut78, SeAx, HH, MOTN-1, KOPT-K1, Jurkat and Loucy were maintained in RPMI-1640 supplemented with 10% FBS, 0.06 g/L penicillin/0.1 g/L streptomycin (Pen/Strep, Gibco, New York, NY, USA), and 2 mM L-glutamine (Gibco). Culture media of KHYG-1, SNK6, MOTN-1 and DERL-2/7 cells was additionally supplemented with 2.5 ng/mL recombinant human IL-2 (ImmunoTools GmbH, Friesoythe, Germany), whereas media of SeAx cells was supplemeted with 5 ng/mL IL-2 and 5 ng/mL IL-4 (ImmunoTools GmbH, Friesoythe, Germany). The culture media for YT cells was Iscove’s Modified Dulbecco’s Medium (IMDM) supplemented with 20% heat inactivated FBS and 20ng/mL human IL-2. The authenticity of the TCL cell lines was confirmed by analysis of highly polymorphic short tandem repeat loci (STR) using the PowerPlex 16 HS System (Promega; performed by Microsynth AG, Dübendorf, Switzerland). Hut78 cells were obtained from CLS Cell Lines Service GmbH, Germany. SU-DHL-1, HH, DERL-2/7, KHYG-1 and, YT cell lines were obtained from the Deutsche Sammlung von Mikroorganismen and Zellkulturen GmbH (DSMZ, Braunschweig, Germany). Jurkat cells were a generous gift from Dr. Florian Grebien (University of Veterinary Medicine Vienna, Vienna, Austria). SNK6 cells were kindly provided by Dr. John Chan (City of Hope Medical Center, Duarte, CA, USA). SeAx and Myla cells were a generous gift from Dr. Keld Kaltoft (University of Aarhus, Aarhus, Denmark). MTA cells were a kind gift from Dr. Raphael Koch (University Medical Center Goettingen, Goettingen, Germany). Mac2a cells were a generous gift from Dr., Marshall Kadin (Brown University, Providence, RI, USA). MOTN-1 cells were kindly provided by Dr. Emmanuel Bachy(Lyon Sud Hospital, Lyon, France). KOPT-K1 cells were a kind gift from Dr. Koshi Akahane (University of Yamanashi, Kofu, Japan). Loucy cells were kindly provided by Dr. A. Thomas Look (Dana-Faber Cancer Institute, Boston, MA, USA). Cell lines were regularly tested for mycoplasma using the MycoAlert mycoplasma detection kit (Lonza Group AG, Basel, Switzerland). All cell lines were cultured at 37 °C in a humidified atmosphere containing 5% CO_2_. Experiments were performed within 20 passages after cell resuscitation. None of the above-mentioned cell lines are listed in the register of cell lines that are known to be misidentified through cross-contamination.

### 4.12. Cytotoxicity Assays

KHYG-1, SNK6, MTA, YT, DERL-2, DERL-7, Mac2a, SU-DHL-1, Myla, Hut78, SeAx, HH, MOTN-1, KOPT-K1, Jurkat, and Loucy cells were plated in 96-well flat-bottom sterile culture plates with low-evaporation lids (Costar #3997). The inhibitors and a vehicle control (0.5% DMSO) were added to the cells following 24 h. After 72 h, Cell Titer-Blue^®^ (Promega #G808A) was added to each well (20 μL), and the fluorescence was measured at 560/590 nm using a Cytation S63 spectrophotometer (BioTek) or on the GloMax^®^ Discover Microplate Reader (Promega, Madison, WI, USA). IC_50_ values were determined using non-linear regression analysis with GraphPad Prism 6.0 (GraphPad Software Inc., San Diego, CA, USA). IC_50_ values represent the effective drug concentration at which cell’s viability is reduced by 50%. IC_80_ concentrations were calculated based on the equation below:

IC(F) = [(100 − F)/F]1/HS × IC_50_

where F = desired percent response (i.e., 80 for 80% reduction in cell viability), HS = Hill Slope.

### 4.13. Synergy Studies

10,000 YT cells/well were plated in a clear, flat-bottom, sterile 96-well plate (Costar #3997) with complete media and incubated overnight at 37 °C and 5% CO_2_. The inhibitors were diluted to 4× starting concentration in complete medium. Inhibitor 1 (at 4× starting concentration) was serial diluted in complete media in a clear, U-bottom, sterile 96-well plate, diluting horizontally (column 3–10). Repeated procedure for inhibitor 2 in a separate 96-well plate, diluting vertically (row A–H). Volume from plate 2 (inhibitor 2) was transferred to plate 1 (inhibitor 1). Bortezomib is diluted to 100 µM (2× starting concentration) in complete media and plated in plate 1 in column 11. Media is plated in control wells (column 2) in plate 1. See Supporting Information Figure S6 for plate layout. Cells were treated with inhibitor combinations from plate 1 for 72h. Wells were treated with Cell Titer-Blue (Promega #G808A) and fluorescence was recorded at 560/590 nm using a Cytation S63 spectrophotometer. A surface plot approach based on the scoring enabled the visualization of the landscape of drug interaction over all the tested dose pairs, providing rich data on the particular dose optimizations that exhibit strong synergy.

## Data Availability

Data is contained within the article or supplementary material.

## Supplementary Materials

The following supporting information can be downloaded at: https://www.mdpi.com/article/10.3390/ph15111321/s1, Scheme S1: Reagents and conditions to synthesize NN-429; Figure S1: Confirms NN-429 fails to cross blood-brain barrier; Table S1: IC_50_ values (μM) calculated from drug response analysis of NN-390, KT-531 and citarinostat; Figure S2: Confirms dose-dependent increase in cells undergoing apoptosis was observed with NN-429 in DERL-7 cells; Figure S3: Table of overall ZIP synergy score and most synergistic area (MSA) score for four separate runs of the NN-429 combinations; Figure S4: Western blot of NN-429 combinations in DERL-7 cells immunoblotted with acetylated α-tubulin, acetylated Histone H3 and HSC70 antibodies; Figure S5: Western blot of NN-429 as a single agent and in combination with SNS-032 in YT cells, cells immunoblotted with acetylated α-tubulin, acetylated Histone H3 and HSC70 antibodies; Figure S6: Synergy plate set-up.
