# Supplementary material for: High Efficacy and Drug Synergy of HDAC6-Selective Inhibitor NN-429 in Natural Killer (NK)/T-Cell Lymphoma
Source: Pharmaceuticals (Basel). 2022 Oct 26;15(11):1321. doi: 10.3390/ph15111321 (PMC9692247; doi:10.3390/ph15111321)

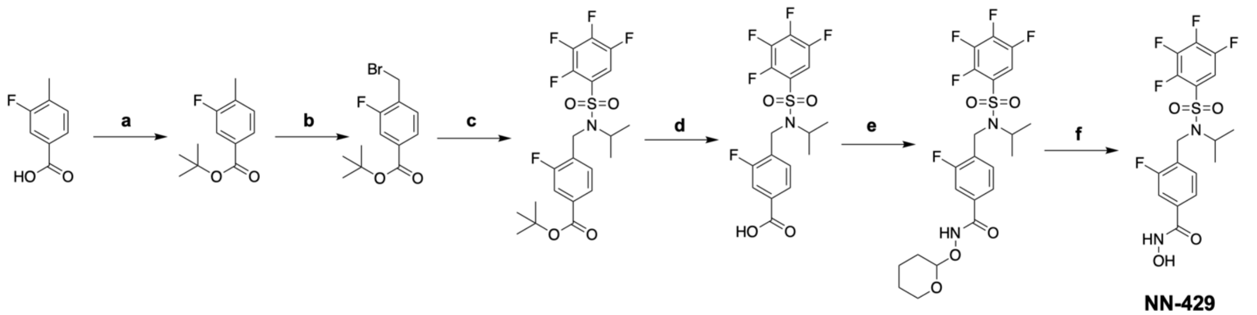

NN-429

Scheme S1. Reagents and conditions to synthesize **NN-429**.

a) ^{t}BuOH, EDC, DMAP, THF, 18 h, RT; b) NBS, AIBN, CCl_4, 4–6 h, 90°C; c) 2,3,4,5-tetrafluoro-*N*-isopropylbenzenesulfonamide, Cs_2CO_3, ACN, 6–18 h, RT; d) 4 M HCl/dioxane, 3–16 h, RT; e) (i) $(COCl)_2$, THF, DMF, 1 h, 0°C; (ii) H_2N-OTHP, $^{i}Pr_2NEt$, THF, 16 h, RT; f) 4 M HCl/dioxane, 3–16 h, RT.

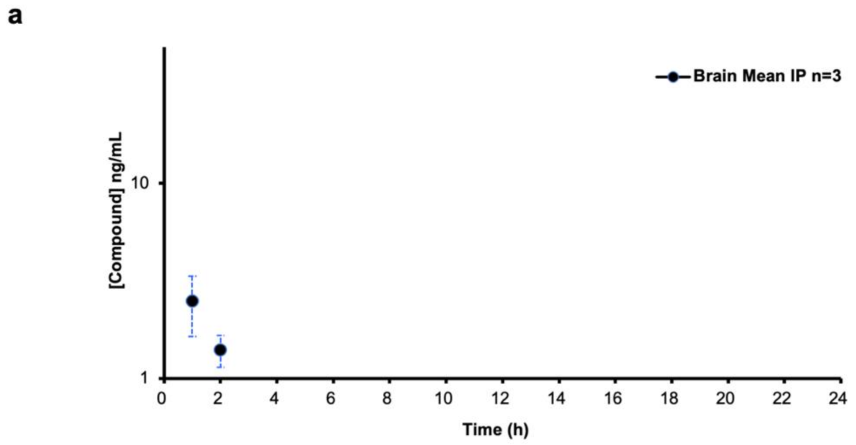

b

Compound	$t_{1/2}$ (h)	C_{max} (ng/mL)	AUC_{last} (h*ng/mL)	AUC_{Inf} (h*ng/mL)	AUC/D (h*mg/mL)
NN-429	NA	NA	NA	NA	NA

Figure S1. *In vivo* brain PK analysis of NN-429. **a.** *In vivo* PK profile of **NN-429** in the brain of male CD-1 mice (n=3) via IP (50 mg/kg). **b.** Mean PK parameters of **NN-429** in the brain of male CD-1 mice (n=3) via IP (50 mg/kg).

Table S1. Cellular cytotoxicity of **NN-429**, **NN-390**, **KT-531** and citarinostat in PTCL malignancies.

Heatmap of IC_{50} values (μM) calculated from drug response analysis of **NN-429**, **NN-390**, **KT-531** and citarinostat from one representative out of 3 independent experiments. Cell lines are classified according to the respective disease subtype. Abbreviations: ANKL, aggressive NK-leukemia; T/NK, T-cell/Natural killer cell; γδ T-NHL, γδ T-cell non-Hodgkin's lymphoma; ALK- ALCL, anaplastic large cell lymphoma (anaplastic lymphoma kinase negative); CTCL, cutaneous T-cell lymphoma.

Compound	Mature TCL				
	ANKL	T/NK cell lymphoma	γδ T-NHL	ALK- ALCL	CTCL
	KHYG-1	SNK6	DERL-2	Mac2a	Myla
**NN-429**	7.25	12.04	2.32	15.07	>50
**NN-390**	3.63	3.1	1.03	7.62	20.23
**KT-531**	3.39	3.53	0.46	2.8	16.17
**Citarinostat**	1.2	2.5	1.5	4.66	3.77

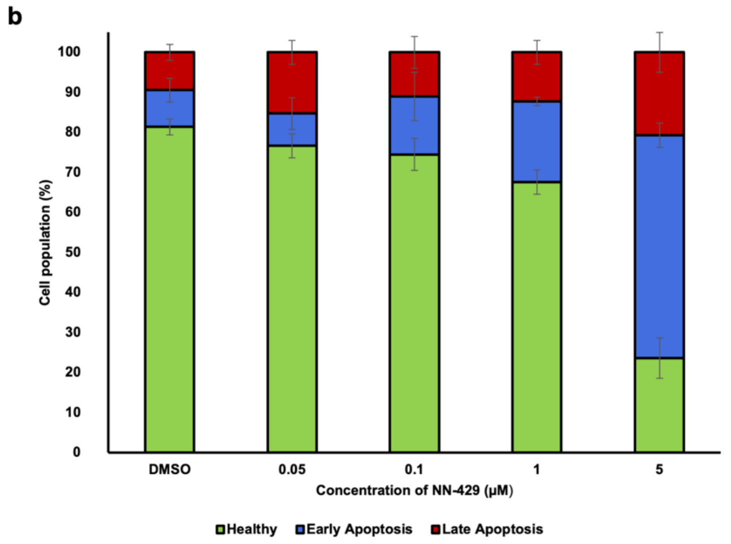

Figure S2. Percentage of DERL-2 cell populations in healthy, early apoptosis, and late apoptosis phases following treatment with **NN-429** for 18 h at the indicated concentrations.

	NN-429 – Doxorubicin		NN-429 – Cytarabine		NN-429 – Etoposide		NN-429 – SNS-032	
	Overall ZIP Synergy Score	Most Synergistic Area Score	Overall ZIP Synergy Score	Most Synergistic Area Score	Overall ZIP Synergy Score	Most Synergistic Area Score	Overall ZIP Synergy Score	Most Synergistic Area Score
Run 1	3.51	12.11	6.99	14.89	7.59	13.97	2.02	5.93
Run 2	1.18	9.78	5.44	8.94	6.41	11.01	1.19	5.73
Run 3	0.90	10.31	3.72	7.15	6.00	12.38	1.61	6.17
Run 4	0.87	6.28	2.13	5.54	5.63	12.91	4.75	11.11
Average	1.61	9.62	4.57	9.13	6.41	12.57	2.39	7.23

Figure S3. Table of overall ZIP synergy score and most synergistic area (MSA) score for four separate runs of the combinations **NN-429** + doxorubicin (dox), **NN-429** + cytarabine (cyt), **NN-429** + etoposide (eto), and **NN-429** + SNS-032 in YT cells.

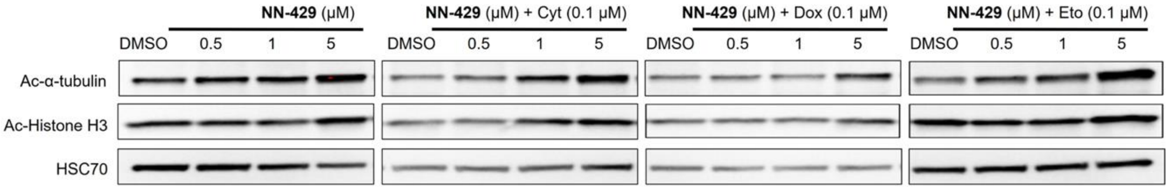

Figure S4. Western blot illustrating α-tubulin acetylation and histone H3 acetylation levels in DERL-7 cells following 24 h treatment with varying concentrations of **NN-429**, **NN-429** + 0.1 µM cytarabine (cyt), **NN-429** + 0.1 µM doxorubicin (dox), **NN-429** + 0.1 µM etoposide (eto). Protein extracts were prepared, resolved by SDS-PAGE and immunoblotted with acetylated α-tubulin, acetylated Histone H3 and HSC70 antibodies were used for loading controls. A representative Western blot of three independent experiments is shown.

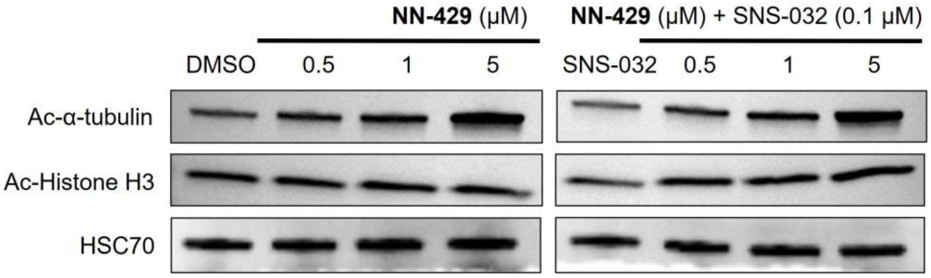

Figure S5. Western blot illustrating α-tubulin acetylation and Histone H3 acetylation levels in YT cells following 24 h treatment with varying concentrations of **NN-429**, **NN-429** + 0.1 µM SNS-032. Protein extracts

were prepared, resolved by SDS-PAGE and immunoblotted with acetylated α-tubulin, acetylated Histone H3 and HSC70 antibodies.

Plate 1

Inhibitor 1 **1/2 Dilution**

	PBS	Medium	Start C.	1/2	1/2	1/2	1/2	1/2	1/2	Inhibitor 2	Bortezomib	PBS
	**1**	**2**	**3**	**4**	**5**	**6**	**7**	**8**	**9**	**10**	**11**	**12**
**A**		Medium	10	5	2.5	1.25	0.625	0.3125	0.15625	0		
**B**		Medium	10	5	2.5	1.25	0.625	0.3125	0.15625	0		
**C**		Medium	10	5	2.5	1.25	0.625	0.3125	0.15625	0		
**D**		Medium	10	5	2.5	1.25	0.625	0.3125	0.15625	0		
**E**		Medium	10	5	2.5	1.25	0.625	0.3125	0.15625	0		
**F**		Medium	10	5	2.5	1.25	0.625	0.3125	0.15625	0		
**G**		Medium	10	5	2.5	1.25	0.625	0.3125	0.15625	0		
**H**		Medium	10	5	2.5	1.25	0.625	0.3125	0.15625	0		

Plate 2

Inhibitor 2 **1/5 Dilution**

	**1**	**2**	**3**	**4**	**5**	**6**	**7**	**8**	**9**	**10**	**11**	**12**	
**A**			10	10	10	10	10	10	10	10			Start C.
**B**			2	2	2	2	2	2	2	2			1/5
**C**			0.4	0.4	0.4	0.4	0.4	0.4	0.4	0.4			1/5
**D**			0.08	0.08	0.08	0.08	0.08	0.08	0.08	0.08			1/5
**E**			0.016	0.016	0.016	0.016	0.016	0.016	0.016	0.016			1/5
**F**			0.0032	0.0032	0.0032	0.0032	0.0032	0.0032	0.0032	0.0032			1/5
**G**			0.00064	0.00064	0.00064	0.00064	0.00064	0.00064	0.00064	0.00064			1/5
**H**			0	0	0	0	0	0	0	0			Inhibitor 1

Figure S6. Plate set-up for synergy studies.

Synthetic Procedures

a) *Tert*-butyl ester protection:

The appropriate benzoic acid was dissolved in THF:*Tert*-butyl alcohol (1:1) mixture (0.1 M in total). 1-Ethyl-3-(3-dimethylaminopropyl)carbodiimide (EDC, 2 equiv.) and 4-Dimethylaminopyridine (DMAP, 2 equiv.) were added and the reactive mixture was stirred for 18 h at RT. The reaction mixture was then diluted in EtOAc and 0.1 M HCl. The organic layer was washed with 0.1 M HCl (1×), water (3×) and brine (1×) and the aqueous layer was extracted once with EtOAc. The combined organic layer was dried over $MgSO_4$, filtered and purified by column chromatography to isolate the target compound.

b) Benzylic bromination:

N-Bromosuccinimide (2.0 equiv.), 2,2'-azobis(2-methylpropionitrile) (AIBN) (0.05 equiv.) and the appropriate tert-butyl protected carboxylic acid (1.0 equiv.) were refluxed in CCl_4 for 10–24 h. The brown mixture was returned to RT and filtered at atmospheric pressure, washed with CCl_4 (2 × 5 mL) and concentrated *in vacuo* to give a brown oil. Column chromatography isolated the purified product.

c) S_N2 substitution with benzyl bromides:

Benzyl bromide (1.1 equiv.) was added to a solution of the amine (1 equiv.) and Cs_2CO_3 (2 equiv.) in ACN (0.1 M). After 6–18 h, the reaction mixture was diluted in EtOAc and saturated aqueous sodium bicarbonate. The organic layer was washed with saturated aqueous sodium bicarbonate (1×), water (3×) and brine (1×) and the aqueous layer was extracted with EtOAc. The combined organic layer was dried over $MgSO_4$, filtered, concentrated *in vacuo* and purified by column chromatography to isolate the target compound.

d) Acid-mediated hydrolysis of carboxylate esters:

The carboxylate or hydroxamate ester was charged in a round-bottom flask with 4 M HCl in dioxane (0.3 M final concentration) at RT in air. After 3–16 h, the solvent was removed *in vacuo*. Carboxylic acid intermediates were used in the next step without further purification.

e) Formation of hydroxamate esters:

Oxalyl chloride (4 equiv.) was added dropwise to a solution of the appropriate carboxylic acid (1.0 equiv.) in THF (0.05–0.2 M) and DMF (1 to 2 drops) at 0°C and stirred for 1–3 h. The reaction was concentrated *in vacuo* before re-dissolving in dry THF (0.2 M) and mixing with diisopropylethylamine or triethylamine (2.0 equiv.) followed by *O*-protected hydroxylamine (1.5 equiv.). After 16 h, the reaction was quenched with 1 M HCl and the layers were separated. The organic layer was washed with 1 M HCl and the combined aqueous layer was extracted with EtOAc or CH_2Cl_2. The organic layer was dried ($MgSO_4$), filtered and concentrated *in vacuo*, and column chromatography isolated the target compound.

f) Acid-mediated hydrolysis of hydroxamate esters:

The carboxylate or hydroxamate ester was charged in a round-bottom flask with 4 M HCl in dioxane (0.3 M final concentration) at RT in air. After 3–16 h, the solvent was removed *in vacuo*. Final hydroxamic acids were purified using preparative HPLC.

Characterization of novel intermediate molecules

3-fluoro-4-(((2,3,4,5-tetrafluoro-*N*-isopropylphenyl)sulfonamido)methyl)-*N*-((tetrahydro-2*H*-pyran-2-yl)oxy)benzamide

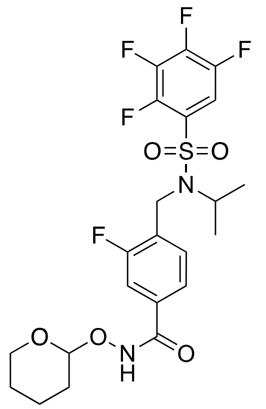

The product was obtained using synthetic procedure (e) as a light yellow oil (67%). ^{1}H NMR (400 MHz, CD_3CN) δ 10.23 (s, 1H), 7.95 – 7.34 (m, 4H), 5.06 – 5.11 (m, 1H), 4.58 (s, 2H), 4.15 – 4.12 (m, 3H), 2.03 – 1.50 (m, 6H), 1.06 (d, *J* = 6.8 Hz, 6H). ^{13}C NMR (101 MHz, CD_3CN) δ 170.7, 163.6, 139.9, 133.7, 130.5, 129.1, 125.7, 125.2, 123.2, 114.07, 112.64, 112.39, 63.3, 60.0, 50.4, 39.1, 39.3, 20.7, 18.3. ^{19}F NMR (376 MHz, CD_3CN) δ -118.17 – -118.73 (m, 1F), -135.57 – -136.53 (m, 1F), -138.05 – -138.73 (m, 1F), -149.09 – -150.02 (m, 1F), -153.42 – -153.78 (m, 1F). LRMS (ESI^+): mass not observed.

tert-butyl 3-fluoro-4-(((2,3,4,5-tetrafluoro-*N*-isopropylphenyl)sulfonamido)methyl)benzoate

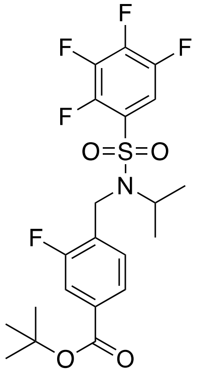

The product was obtained using synthetic procedure (c) as a white solid (54%). ^{1}H NMR (400 MHz, $CDCl_3$) δ 7.76 – 7.72 (m, 1H), 7.63 – 7.60 (m, 1H), 7.56 – 7.53 (m, 1H), 7.51 – 7.41 (m, 1H), 4.58 (s, 2H), 4.22 (p, *J* = 6.8 Hz, 1H), 1.59 (s, 9H), 1.30 – 0.96 (m, 6H). ^{19}F NMR (376 MHz, $CDCl_3$) δ -118.50 – -119.03 (s, 1F), -134.03 – -134.93 (m, 1F), -135.92 – -136.44 (m, 1F), -146.82 – -148.53 (m, 1F), -151.44 – -151.97 (m, 1F). ^{13}C NMR (101 MHz, $CDCl_3$) δ 164.1, 164.1, 145.1, 144.5, 143.1, 141.2, 140.0, 139.8, 139.2, 133.7, 116.09, 115.5, 112.2, 81.6, 50.4, 39.1, 28.1, 20.9. LRMS (ESI^+): mass not observed.

tert-butyl 4-(bromomethyl)-3-fluorobenzoate

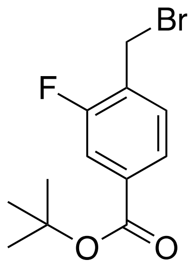

The product was obtained using synthetic procedure (b) as a brown oil (40%). ^{1}H NMR (400 MHz, CD_3CN) δ 7.64 – 7.62 (m, 1H), 7.56 – 7.51 (m, 1H), 7.26 (m, 1H), 4.58 (d, *J* = 1.0 Hz, 2H), 1.58 (s, 9H). ^{13}C NMR (101 MHz, CD_3CN) δ 164.3, 159.9, 131.1, 131.4, 129.8, 124.8, 80.3, 27.4, 20.8. ^{19}F NMR (376 MHz, CD_3CN) δ -111.81 – -122.48 (m, 1F). LRMS (ESI^+): mass not observed.

tert-butyl 3-fluoro-4-methylbenzoate

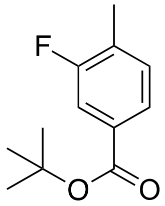

The product was obtained using synthetic procedure (a) as a clear oil (67%). ^{1}H NMR (400 MHz, CD_3CN) δ 7.64 – 7.62 (m, 1H), 7.55 (d, *J* = 10.5, 1H), 7.25 – 7.22 (m, 1H), 2.29 (s, 3H), 1.58 (s, 9H). ^{19}F NMR (376 MHz, CD_3CN) δ -105.05 – -130.54 (m, 1F). ^{13}C NMR (101 MHz, CD_3CN) δ 164.3, 159.7, 131.4, 124.9, 124.0, 117.0, 115.3, 80.3, 22.3, 20.6. LRMS (ESI^+): mass not observed.

Characterization of final compound

NN-429

3-fluoro-*N*-hydroxy-4-(((2,3,4,5-tetrafluoro-*N*-isopropylphenyl)sulfonamido)methyl)benzamide

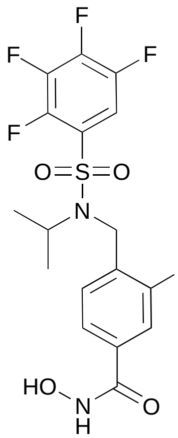

The product was made using synthetic procedure (f), followed by preparative HPLC and lyophilization to obtain a white powder (39%). ^{1}H NMR (400 MHz, Acetone) δ 10.85 (s, 1H), 7.88 – 7.40 (m, 4H), 4.64 (s, 2H), 4.47 – 4.18 (m, 1H), 1.10 (dd, J = 6.8, 3.0 Hz, 6H), *Hydroxamic acid OH proton was not observed.* ^{19}F NMR (376 MHz, MeOD) δ -118.98 – -119.12 (m, 1F), -136.34 – -137.10 (m, 1F), -138.56 – -139.80 (m, 1F), -150.04 – -151.11 (m, 1F), -154.73 (d, J = 19.7 Hz, 1F). ^{13}C NMR (126 MHz, acetone) δ 161.8, 160.6, 149.0, 146.1, 144.1, 143.2, 132.1, 131.6, 131.5, 126.4, 123.7, 122.6, 113.5, 112.8, 51.4, 40.5, 22.7. HRMS (ESI^{+}) m/z calcd for [$C_{17}H_{16}F_{5}N_{2}O_{4}S$]+: 439.0745, found: 439.0710. HPLC (I) t_R = 34.42 min; HPLC (II) t_R = 36.77 min (99%).

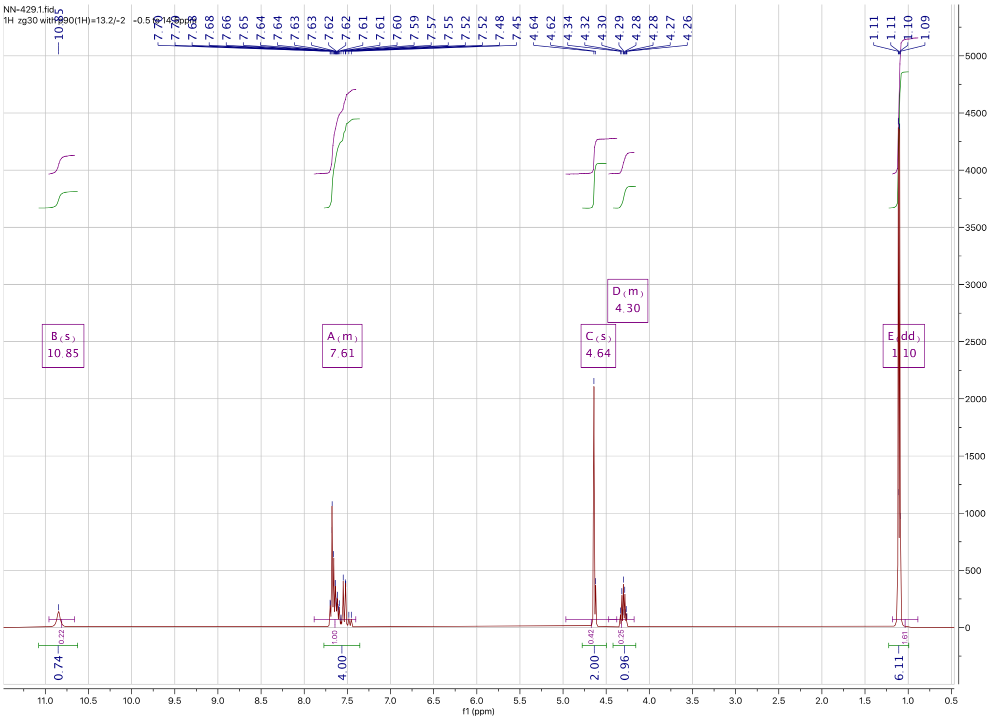

Supplement: Supplementary file 1 [file pharmaceuticals-15-01321-s001.zip › pharmaceuticals-1894599-supplementary.pdf]
